# Reusable 3D-printed microfluidic-on-fabric with modular electrodes for point-of-care Na^+^ and K^+^ detection in various biofluids

**DOI:** 10.1007/s00216-026-06569-0

**Published:** 2026-05-22

**Authors:** Tao Zhang, Grayson Ruffner, Sarah Varney, Inchan Baek, Bella Fong, Hui Chen, Chengpeng Chen

**Affiliations:** 1 Department of Chemistry and Biochemistry, University of Maryland Baltimore County, 1000 Hilltop Circle, Baltimore, MD 21250 USA; 2 Department of Chemistry and Biochemistry, James Madison University, 901 Carrier Drive, Harrisonburg, VA 22801 USA; 3 Phillips Academy, Andover, MA 01810 USA

**Keywords:** Point-of-care, Sensor, Microfluidics, 3D-printing

## Abstract

In this study, we present a new 3D-printed microfluidic-on-fabric sensor platform that allows for point-of-care (POC) detection in various biofluids. Using the low-cost FDM 3D-printing, we developed a method to form well-defined microfluidic features on fabric substrates (3D-printed microfluidic-on-fabric), which could modularly house an electrode pad with desired electrodes targeting different analytes. The 3D-printed microfluidic-on-fabric integrates the comfort, robustness, and reusability (after washing) of fabric substrates with the precision, modularity, and scalability offered by 3D-printed structures. We showcased the application of the sensor platform for Na⁺ and K⁺ detections with lab-made ion-selective electrode pads. The electrodes showed high reusability, in addition to the robust Nernst sensitivity, accuracy, selectivity, and reproducibility (linear range = 5–500 mM; LOD(Na⁺) = 1.5 mM and LOD(K^+^) = 1.6 mM). The sensor platform was successfully implemented to detect Na⁺ and K⁺ in a range of biofluids, including urine, saliva, and sweat, with results validated by ion chromatographic methods. An Arduino board (~ $20) was programmed to couple to the sensor for data collection, transducing, processing, and transferring to a computer. Overall, this is a new sensor platform that is low-cost, reusable, modular, and customizable, representing an advancement in POC sensor development.

**Supplementary Information:**

The online version contains supplementary material available at 10.1007/s00216-026-06569-0.

## Introduction

Point-of-care (POC) diagnostics have become increasingly vital in healthcare due to their ability to provide rapid, accessible, and cost-effective testing outside of centralized laboratories [1, 2]. These decentralized POC tools enable timely clinical decisions, improve patient outcomes, and are especially valuable in resource-limited settings or for patients requiring frequent monitoring of chronic conditions [3]. The demand for POC technologies has grown significantly as healthcare systems shift towards personalized and preventive care [4–6].

There is an increasing demand for simple-to-use, accurate, and non-invasive methods to detect sodium (Na⁺) and potassium (K⁺) ions in a range of biofluids, as these electrolytes are critical biomarkers [7]—imbalanced Na⁺ and K⁺ concentrations are strongly linked to conditions such as hypertension, dehydration, electrolyte disturbances, and cardiovascular diseases, highlighting the clinical significance of rapid and frequent monitoring of the ions [8]. In biofluids such as urine, Na⁺ and K⁺ measurements provide valuable insights into kidney performance and dietary electrolyte intake; in saliva, they offer a non-invasive reflection of systemic electrolyte status [9]; and in sweat, they enable quick monitoring of electrolyte loss during exercise, heat exposure, or illness. The ability to accurately track Na⁺ and K⁺ levels across these matrices is essential for early diagnosis, personalized care, and improved treatment outcomes [10].

Microfluidic systems have been widely applied for POC detections due to the controllable sample delivery and the potential for multiplexing among channels and reservoirs, particularly the paper-based microfluidics, which requires fewer/cheaper facilities to fabricate and prepare than soft-lithography-based microfluidic processes, and can actively deliver liquid samples via capillary action [11]. Nonetheless, paper substrates are inherently fragile, which can limit their reusability and long-term applicability [12–14]. On the other hand, fabric-based microfluidics are emerging as promising apparatus, especially for applications where the microfluidic device needs to contact human skin, offering enhanced comfort, flexibility, durability, and broad compatibility with various detection strategies [15, 16]. In the past decade, three-dimensional (3D) printing has reshaped the development of lab devices by enabling rapid prototyping, customizable designs, intricate 3D structures, and shareable device designs [17–22], facilitating scalable manufacturing of microfluidic devices for various applications, including POC diagnostics [23–27]. Fused deposition modeling (FDM)–based 3D printers especially attract much research interest due to the low cost (a printer typically below a thousand US dollars; filament several cents per foot) [28, 29].

In this work, we present a new electrochemical POC sensor platform combining 3D-printed microfluidic features and cotton fabric substrates. This platform showed impressive structural robustness and reusability (reusable for ~ 60 cycles with vigorous washing). Meanwhile, a slot was 3D-printed at the end of the microfluidic channel for click-assembling and disassembling the electrodes, which were screen-printed on a matching 3D-printed pad. Electrodes are typically reusable after rinsing in electrochemical detections, so were ours validated by experimental results. These features can further reduce the cost of such sensors and thus enhance translation, especially in underdeveloped areas or for patients with a high medical burden already. This platform will also allow for the potential formation of a kit where the reusable 3D-printed microfluidic-on-fabrics can accommodate different electrode pads tailored to individual health monitoring needs. Here, we showcased the applicability of this sensor platform for Na^+^ and K^+^ because of the importance of the ions as biomarkers mentioned above. Results showed successful, accurate, and reproducible Na^+^ and K^+^ detections from various biofluids: urine, saliva, and sweat. An Arduino board was programmed and connected to the electrodes as the transducer and readout device, the low cost of which (~ $20) can further facilitate technology translation for POC ion detections.

## Experimental

### Chemicals and materials

Tetrahydrofuran (THF), poly(vinyl chloride) (PVC), bis(2-ethylhexyl) sebacate (DOS), sodium tetrakis [3,5-bis(trifluoromethyl)phenyl] borate (Na-TFPB), sodium ionophore X, valinomycin, sodium tetraphenylborate (Na-TPB), ethanol, and acetone were purchased from MilliporeSigma (MO, USA). Sodium chloride (NaCl), isopropanol, and methanol were purchased from Thermo Fisher Scientific (MA, USA). Ammonium chloride (NH_4_Cl) was purchased from Alfa Aesar (MA, USA). Potassium chloride (KCl) was purchased from Research Products International (IL, USA). Mineral oil was obtained from Ward’s Science (NY, USA). Polyurethane (PU, SG-80a) was sourced from Lubrizol (OH, USA). Multiwalled carbon nanotubes (MWCNTs) were acquired from Tokyo Chemical Industry (Tokyo, Japan). Polyvinyl butyral (PVB) was purchased from ChemCruz (CA, USA). Silver/silver chloride (Ag/AgCl) paste was obtained from Ercon (MA, USA). Silver conductive epoxy adhesive was acquired by MG Chemicals (ON, CA).

### 3D-printing microfluidics

**Fig. 1:**
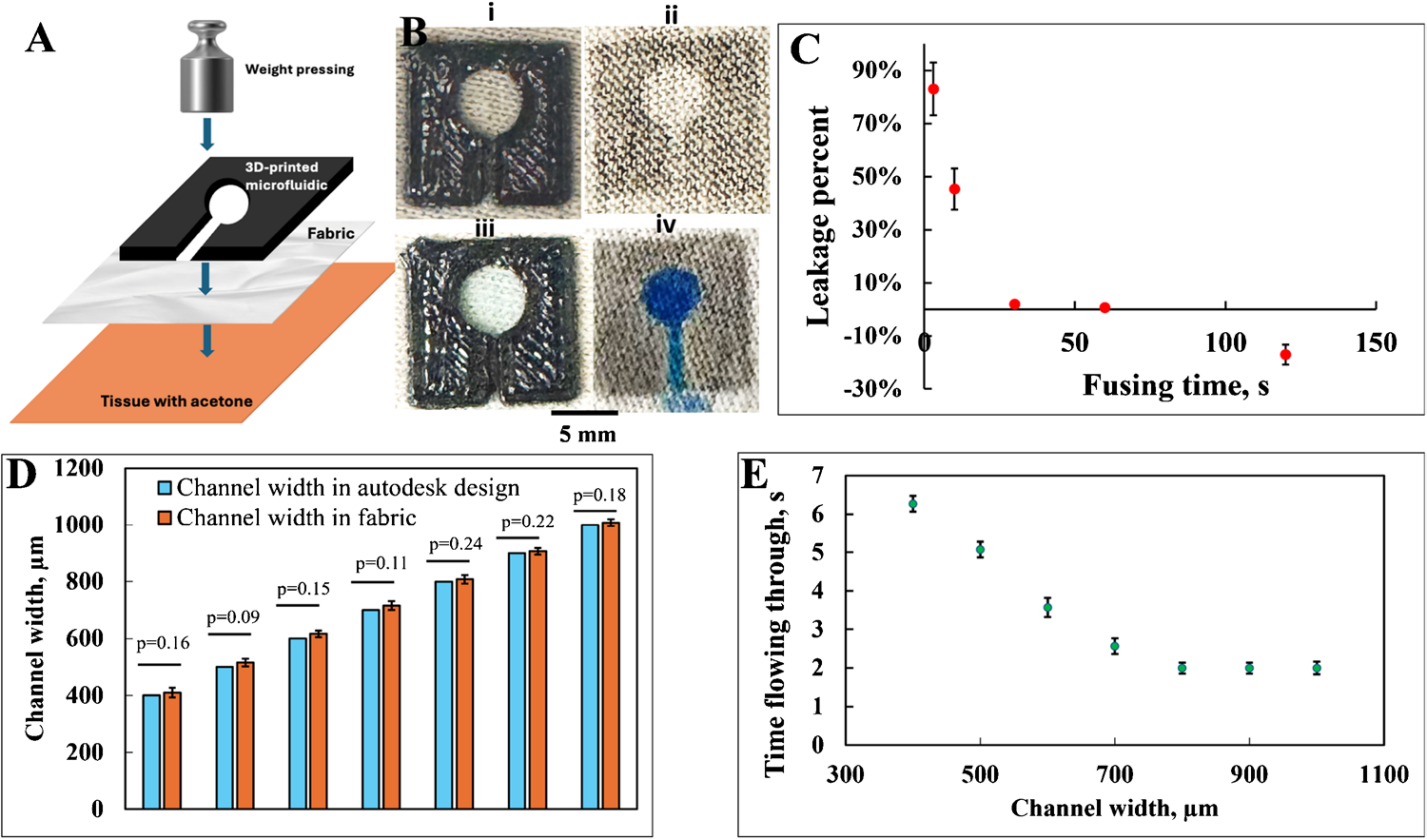
The technology to make 3D-printed microfluidics-on-fabric, and device characterization. **A** Illustration of the fabrication process. The acetone in the tissue vaporized and diffused up through the fabric layer, partially dissolving the 3D-printed ABS part, which then infiltrated down into the fabric, creating hydrophobic barriers. **B** Front (i)/back (ii) images of an example device; (iii) and (iv) show the device loaded with a blue dye solution, front and back. **C** Characterization of possible leakage of the microfluidic-on-fabric device as a function of layer-fusing time. (*N* = 3, error bar = standard deviation). **D** Comparison of device channel widths to CAD design widths (*N* = 3, error bar = standard deviation). **E** Time needed for a dye loaded to the reservoir to flow out the channel of various widths. (*N* = 3, error bar = standard deviation)

**Fig. 2:**
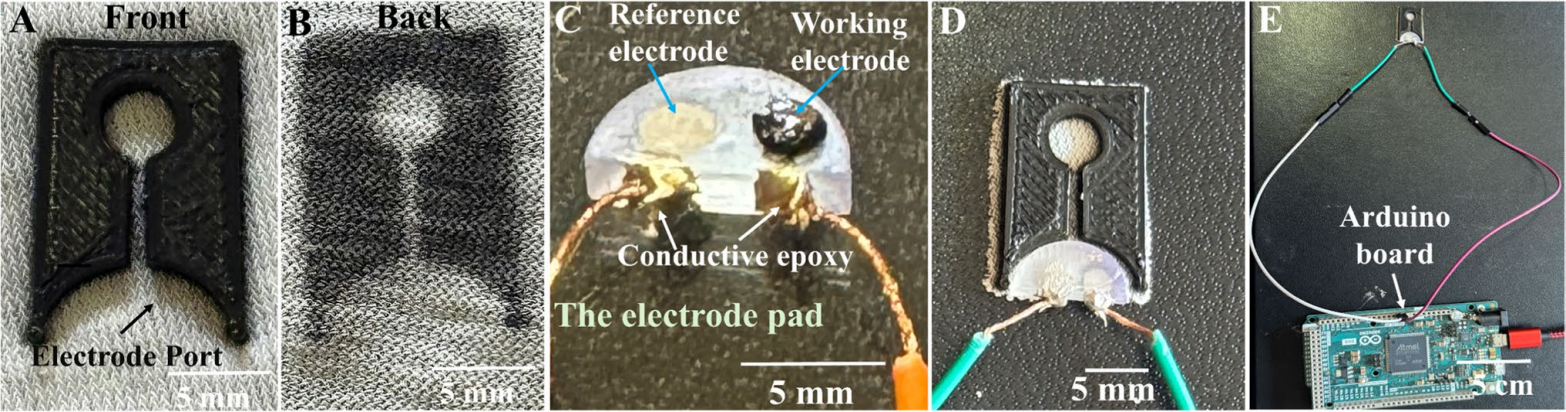
**A**, **B** Front and back view of the sensor microfluidic-on-fabric. **C** The electrode pad with a reference and working (ion-selective) electrode connected to wires via conductive epoxy. The electrodes will face down contacting the fabric when being assembled to the microfluidic device’s port. **D** Picture of an assembled device. **E** The sensor was connected to an Arduino board, which was programmed as a transducer for ion-selective readings

Microfluidic structures were designed using Autodesk Inventor Professional 2026 and featured a rectangular base (various sizes, details in Figs. 1 and 2), with a circular reservoir, various microchannel widths, and a semicircular port, with the overall device’s height of 2 mm. The design was exported and converted into a.gx file using FlashPrint5 slicing software. Structures were printed using a FlashForge Adventurer 4 Pro 3D printer with acrylonitrile butadiene styrene (ABS) filament (purchased from Amazon). Printing parameters included a nozzle temperature of 240 °C and a heated platform set to 110 °C.

To fuse a 3D-printed microfluidic structure onto a fabric substrate, a piece of paper towel was soaked in acetone, on top of which a fabric layer was laid, and then the microfluidic piece was placed atop the fabric. A 200 g weight (those to calibrate a mechanical balance) was applied on top of the paper towel-fabric-3D-printed microfluidic sandwich with the help of a glass plate to enhance the contact of the layers. Various pressing time durations were tested to find the optimal.

### The sodium- and potassium-selective electrodes

A semicircular electrode base was designed using Autodesk Inventor Professional 2026 as semicircles to fit the semicircular port of the microfluidic device. The part was then 3D-printed using a Formlabs 3D printer with the BioMed Clear V1 resin. Printed parts were rinsed in isopropanol for 30 min and cured under UV and heat using a Form Cure system for 60 min.

A stencil laser cut from a polyester film was created to print dot-shaped electrodes onto the semicircular electrode base. The stencil was designed in CorelDRAW 2018 with two 2-mm holes. The stencil was aligned with the 3D-printed electrode base, and Ag/AgCl ink was squeezed across the stencil on the semicircular electrode base, followed by air drying at room temperature, so that two electrode dots were formed. Additional Ag/AgCl paste was hand-applied to the flat edge of the semicircle to provide a wire connection point on the vertical plane of the pad.

The reference Ag/AgCl electrode dots were coated with 3 μL of a solution containing 78 mg polyvinyl butyral (PVB) and 50 mg NaCl in 1 mL methanol to serve as the reference electrode. The working electrode also used Ag/AgCl as the base, coated by an ion-selective membrane. The Ag/AgCl base (in a dot shape) was first coated by 3 μL carbon nanotube ink composed of 0.10 g multiwalled carbon nanotubes (MWCNTs), 0.5 g polyurethane (PU), and 180 μL mineral oil dissolved in 3 mL tetrahydrofuran (THF). The potassium-selective membrane was prepared by pipetting 3 μL of a membrane cocktail containing 2% valinomycin (K⁺ ionophore), 0.5% sodium tetraphenylborate (NaTPB), 32.7% PVC, and 64.7% bis(2-ethylhexyl) sebacate (DOS) in 660 μL THF, on top of the air-dried MWCNT coating, followed by air drying. The sodium-selective membrane followed the same procedure with the coating cocktail containing 1% Na ionophore X, 0.55% sodium tetrakis [3,5-bis(trifluoromethyl)phenyl] borate (Na-TFPB), 33% PVC, and 64.45% DOS dissolved in 660μL THF. Electrical connections were made by attaching jumper wires to the electrodes using a silver-based conductive epoxy adhesive [20, 30–33].

### The Arduino transducer

An Arduino DUE board was used in conjunction with the free, open-source Arduino IDE software to measure ion-selective potential responses (https://docs.arduino.cc/programming/). A custom program was coded to record the voltage between the ion-selective and reference electrodes. The reference electrode was connected to port A0 of the Arduino board, serving as ground. CoolTerm was used to interface with the Arduino to a laptop for real-time data collection and data exporting to Microsoft Excel for analysis.

### Characterization of the ion-selective electrodes

#### Calibration curves

Standard solutions of Na⁺ and K⁺ were prepared at the following concentrations: 5 mM, 10 mM, 50 mM, 100 mM, and 500 mM using doubly deionized (DDI) water. For each standard, 100 µL was pipetted to the reservoir of a microfluidic device, then measured by the electrodes click assembled to the end of the fluidic channel, with voltage signals read and recorded by the Arduino. The voltage responses were then plotted as a function of the standard concentrations (log scale).

#### Accuracy/recovery tests

Na⁺ and K⁺ solutions were prepared within the physiological ranges: 50 mM and 200 mM Na⁺, and 25 mM and 100 mM K⁺, made in DDI water. Each solution was measured three times with three sensor devices (microfluidic-on-fabrics + the electrode pad). The solutions were also measured via ion chromatography (IC). The sensor readouts, the IC results, and the analytical/recipe concentrations were then statistically compared.

#### Selectivity tests

Selectivity was tested by comparing electrode responses in the presence of common monovalent cations found in human biofluids. For the sodium electrode, three solutions were tested: 200 mM Na⁺, 200 mM Na⁺ + 200 mM NH₄⁺, and 200 mM Na⁺ + 200 mM K⁺. For the potassium electrode, the tested solutions were 25 mM K⁺, 25 mM K⁺ + 25 mM NH₄⁺, and 25 mM K⁺ + 25 mM Na⁺.

#### Reusability tests

To test the structural integrity of the microfluidic-on-fabric devices, identical ABS-based 3D-printed microfluidic structures (one reservoir, one channel) were fused separately on paper (for comparison) or fabric substrates. Each device was subjected to repeated washing cycles by pipetting 5 mL 50/50 water/ethanol (V/V) mixture into the reservoir to rinse the device, and structural integrity was compared by monitoring leakage of a loaded food dye solution out of the microfluidic channel/reservoir over time.

To test the reusability of the Na^+^-selective electrode module, 10 μL of sodium solutions made in DDI water (100 mM) were pipetted into the sensor reservoir, followed by voltage readings. Then, 5 mL 50/50 water-ethanol solution (V/V) was pipetted to the electrodes for washing. After being dried, the electrodes were used again to measure the same Na^+^ solution. The same test was also performed for the K^+^-selective electrode by using a 25 mM potassium solution.

To test if the washed/reused microfluidic-on-fabric devices may cause sample carryover issues, one hundred microliters sodium solution made in DDI water (100 mM) was repeatedly measured. The loaded solution to the reservoir was wicked through the microfluidic channel and measured by electrodes assembled at the channel outlet. This process was repeated with the same devices after washing with 5 mL 50/50 water-ethanol (V/V, into the reservoir then allowed to flow out), in order to assess potential carryover effects across reusing cycles.

### Ion chromatographic (IC) measurement of Na⁺ and K⁺

A CS16 column purchased from Thermo Fisher was used for the measurements. The Thermo Scientific Dionex ICS-6000 HPIC system (Middletown, VA) was used, coupled with the Dionex ICS-6000 SP single pump and DP dual pump, and the Thermo Scientific™ Dionex™ ICS-6000 CD conductivity detector. The eluent was delivered at a constant flow rate of 1.0 mL/min, using an isocratic method with 39 mM methanesulfonic acid. Calibration curves were generated by plotting the integrated peak area against the corresponding concentration for each standard. For samples, they were filtered by syringe filters (pore size, 0.22 μm; diameter, 33 mm) and then diluted 10 times using DDI water before IC analysis. The concentrations of Na⁺ and K⁺ in biofluid samples were then quantified using the calibration curves. The calibration curve and chromatograph examples are shown as Figures S1 and S2.

### Real application of the sensor to measure the ions from various biofluids

The UMBC IRB protocol #1343 was followed to collect sweat from human skin surface. Simulated human saliva were purchased from Biochemazone (Canada). For urine and saliva tests, a sample was dispensed into the reservoir of a microfluidic device by a pipette, which then flowed through the microfluidic channel to the electrode pad for sensing. For the sweat test, the microfluidic device was held tilted with the reservoir area touching a sweating surface. Voltages were recorded and processed by the Arduino Coding (Figure S3), with sodium and potassium concentrations reported on a laptop screen, as well as the sodium-to-potassium ratio. All sensor results were validated by measuring the samples via ion chromatography as aforementioned.

### Data analysis and statistics

Replicate numbers and error bars are presented in each figure. ANOVA and *t*-test were applied to compare data groups, and a significant difference was determined only when a *p*-value was smaller than 0.05. The limit of detection (LOD) was determined using the noise-based approach. Blank samples containing no analyte were measured repeatedly under identical conditions, and the standard deviation of the resulting signal was calculated. The LOD was defined as three times the standard deviation (3σ) plus the blank signal.

## Results and discussion

### The 3D-printed microfluidic-on-fabric devices

FDM 3D-printing is low cost in both the printer and materials, and recent advances can make such printers’ resolution to sub-100 µm. Pioneering works have also suggested the feasibility of printing microfluidic devices with FDM printers [34–40]. Acrylonitrile butadiene styrene (ABS) is a common FDM printing material that is low-cost and durable. More importantly, ABS dissolves in mild solvents such as acetone [41]. To make microfluidics on a wicking flat substrate, such as paper and fabric, the critical step is to form hydrophobic barriers completely through the substrate to define channels and reservoirs in the middle [42–44]. We therefore explored fusing 3D-printed microfluidic patterns onto cotton fabrics using acetone. As noted above, both 3D-printed microfluidics and fabric substrates offer unique advantages, and their integration could enable new applications. As shown in Fig. 1A, a 3D-printed ABS microfluidic part (black) was placed on a piece of cotton fabric (white), which was then positioned on a piece of paper towel saturated with acetone in a sandwiched arrangement. A weight was applied on top to ensure close contact. The acetone vapor going up through the fabric layer could partially dissolve the ABS layer, which then infiltrated down into the fabric, creating hydrophobic barriers. Figure 1B presents a single-channel device formed this way. We found that fusion time was a critical parameter: insufficient time hindered ABS infiltration, whereas excessive time caused lateral spreading of dissolved ABS, leading to channel distortion or blockage. A blue dye solution was added to the reservoir and allowed to flow through the channel. The dyed region on the opposite side of the fabric was quantified using ImageJ. A dyed area larger than the designed channel/reservoir indicated incomplete ABS infiltration, thereby liquid leakage (positive leakage), whereas a smaller area suggested channel or reservoir shrinkage (negative leakage), likely caused by lateral diffusion of dissolved acetone. As shown in Fig. 1C, when fusion time was less than 30 s, extensive leakage of the dye solution occurred beyond the channel and reservoir, indicating that robust hydrophobic barriers had not yet formed. Between 30 and 60 s, a stable structure was achieved, and leakage was eliminated. Beyond 60 s, the dyed area became smaller than designed, suggesting channel and reservoir shrinkage. These results indicate that a treatment time of 30–60 s is optimal for fabricating the 3D-printed microfluidics-on-fabric.

Next, we characterized if the final channel sizes were off from the CAD design sizes, and if so, to what degree. We 3D-printed microfluidic parts like the one in Fig. 1B, with channel widths ranging from 400 to 1000 µm, a typical range for POC sensor microfluidics [45, 46]. As shown in Fig. 1D, no significant differences were detected between the design channel widths and the final widths on the devices. Since the device delivers liquid through capillary action within the cotton yarn fibers, the microfluidic channel width determines the effective flow flux area and thereby influences volumetric flow rates. To characterize that, we pipetted a dye solution into the reservoir, recorded the time needed to flow out of the channel, and plotted the data in Fig. 1E. Between 800 and 1000 µm, no significant differences were observed.

These findings demonstrate that 3D-printed microfluidic patterns can be reliably fused onto cotton fabric substrates with well-defined channel widths and consistent liquid delivery. Optimal performance was achieved with a fusing time of 30–60 s and channel widths larger than 800 µm, which enabled the most efficient liquid transport.

### The sensor microfluidic design and the modular electrode pad

As shown in Fig. 2A, B, a microfluidic pattern was 3D-printed from black ABS filament and then fused to a white cotton fabric layer. The round reservoir on top was where a sample was loaded/collected. Through the channel, the end was a semicircular port to house an electrode pad. Figure 2C shows an electrode pad with dot-shaped reference and working (ion-selective) electrodes printed on a 3D-printed semicircular structure and the electrodes connecting to lead wires [11]. Figure 2D is a picture of an assembled device with an electrode pad click-assembled into the ending port of the microfluidic; the electrode side faced down onto the fabric, so that liquid delivered from the channel could touch the electrodes for readings, transduced by the programmed Arduino board (Fig. 2E).

Previous studies have shown that microfluidic structures are necessary in point-of-care devices as the orderly directed flow (typically via capillary actions) can enhance detection sensitivity and reduce sample consumption [47, 48]. A key advantage of this 3D-printed microfluidic-on-fabric platform lies in its modularity. Our ultimate goal is to have a kit with some microfluidic-on-fabric devices and an array of electrode pads targeting different markers, so that the kit can be used in a customizable way for a broad range of health monitoring in a POC setting. In this paper, we will discuss Na^+^ and K^+^ ion-selective electrodes. Moving towards that goal, the robustness and reusability of the microfluidic devices and the electrodes need to be known.

**Fig. 3:**
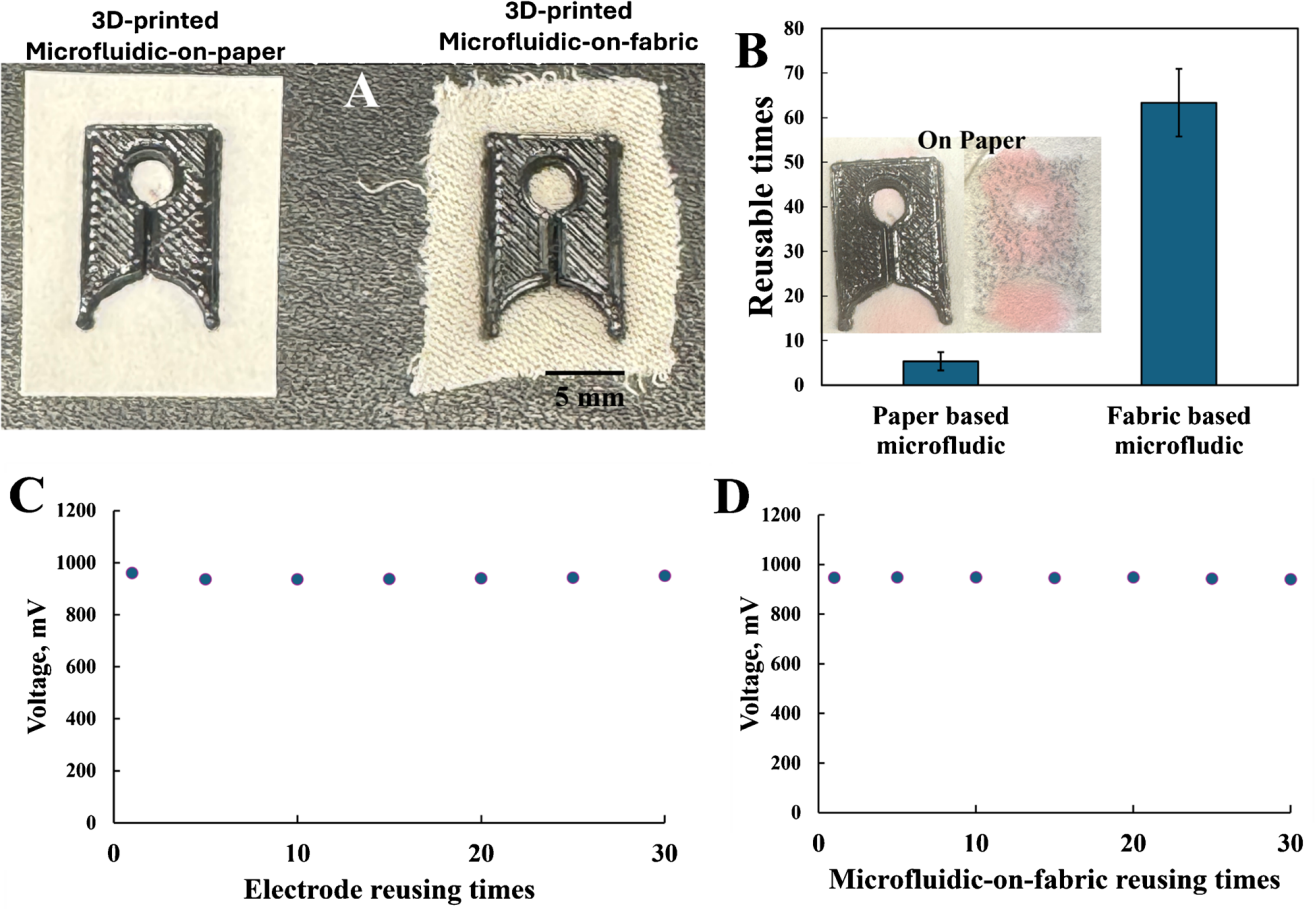
The sensor reusability evaluation. **A** 3D-printed microfluidic-on-paper (left) and microfluidic-on-fabric (right). **B** Reuse time limits (before structural damage) for the microfluidic devices. Inset is the front and back view of a 3D-printed microfluidic-on-paper, after seven times of reusing. The back view showed significant leakage of the dye solution out of the microfluidic features. **C** Reusability test of the ion-selective electrode, with rinsing after each use. **D** Characterization of possible sample carryover. A high concentration (100 mM) of Na^+^ was loaded to the microfluidic reservoir, then voltage read by the ion-selective electrode, followed by washing the device and repeating the measurements. Results did not show carryover from prior samples

As shown in Fig. 3A, we could fuse the 3D-printed microfluidic (ABS) features onto paper substrates with the same method. We then pipetted a dye solution into the microfluidic reservoir and let it flow out of the channel, followed by vigorously pipetting 5 mL 50/50 water/ethanol mixture to rinse the device. This process was defined as one cycle. As shown in Fig. 3B, the paper-based microfluidics exhibited structural failure and leakage out of the channel and reservoir after a few uses (5.3 ± 2.0 times, mean ± standard deviation). In contrast, the fabric-based devices demonstrated significantly greater durability, maintaining stable performance for 60–70 reuse cycles. Because ion-selective electrodes are typically reusable in research and testing laboratories, we therefore explored how reusable our lab-made ones were on the sensor. As shown in Fig. 3C, the Na^+^-selective electrode maintained consistent voltage responses using a 100 mM Na^+^ solution across 30 cycles (with water/ethanol rinsing in between), indicating that the electrode could be reused for at least 30 times. Similar reusability was also found for the K^+^-selective electrode.

Having established the structural robustness of the 3D-printed microfluidic-on-fabric, we next asked whether device reuse could lead to sample carryover. We kept reusing a device (the microfluidic-on-fabric + the electrode pad) to measure a Na^+^ standard. After each measurement, 5 mL of 50/50 water/ethanol was vigorously pipetted to the microfluidic reservoir to rinse the channel and the downstream electrodes, followed by air drying for 30 min. As shown in Fig. 3D, readings from the repetitive measurements remained stable over 30 measurements, without significant drift of the voltage readings, indicating that at least for ion detections, there were no observable carryover problems from old samples.

The data in Figs. 2 and 3 suggest that this 3D-printed microfluidic-on-fabric represents a promising POC sensor platform, combining robust structures with high reusability and supporting modular click-assembly of electrodes for desired detections.

### Applying the sensor for Na^+^ and K^+^ detection from various biofluids

#### Validation of the sensor’s analytical performance

**Fig. 4:**
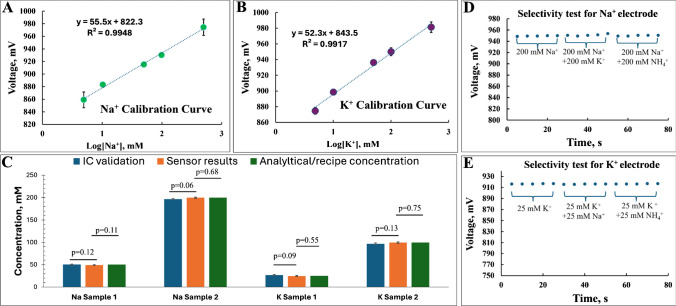
Analytical performance of the sensor. **A** Na^+^ calibration curve. *N* = 3, error bar = standard deviation. **B** K^+^ calibration curve. *N* = 3, error bar = standard deviation. **C** Sample recovery results and statistical comparisons with the validation method (ion chromatography, IC). *N* = 3, error bar = standard deviation. **D**, **E** Selectivity tests of the ion-selective electrodes

First, Na⁺ and K⁺ standard solutions in physiological ranges (5 mM to 500 mM) [7, 49] were prepared, measured, and plotted, as shown in Fig. 4A, B, both Na⁺ and K⁺ readings exhibited linear correlations between the potentials building up on the electrode surfaces and the logarithm scale of the ion concentrations. The LOD of Na⁺ and K^+^ electrodes were determined to be 1.5 mM and 1.6 mM, respectively. The slopes, 55.5 mV for Na⁺ and 52.3 for K⁺, were highly consistent with the Nernst equation (theoretical slope for monovalent cations is 59.2 mV) [50, 51].

To validate accuracy of the sensors, sample recovery was conducted. As shown in Fig. 4C, two Na⁺ solutions (50 mM, 200 mM, physiologically relevant) were tested by the sensor, and the detected values (orange bars) were consistent with the analytical/recipe concentrations (green bars). We further validated the accuracy by also measuring the solutions by ion chromatography (blue bars), a gold standard lab method for ion quantification[52–57], and did not find statistical differences between the sensor and ion chromatography results.

Selectivity was also evaluated by mixing common monovalent cations in biofluids that are in large quantities (Na⁺, K⁺, and NH₄⁺). For the Na⁺ sensor (Fig. 4D), no interference was observed from K⁺ and NH_4_⁺, and the K⁺ sensor (Fig. 4E) also showed non-detectable interferences from the possible interfering ions.

Reproducibility of the sensors was also elucidated from the data. All error bars were standard deviations. From the calibration curves, the average relative standard deviation (RSD) was 0.39% for Na^+^ and 0.75% for K^+^ using three different electrodes/sensors. From the recovery experiments, the average RSD for the Na^+^ sensor was 1.50%, compared to 1.54% of ion chromatography. The RSD for the K^+^ sensor was also smaller than the ion chromatographic method (1.51% vs. 2.06%). These results validated the analytical robustness of the sensors for the ion detections.

#### Test of various biofluids

**Fig. 5:**
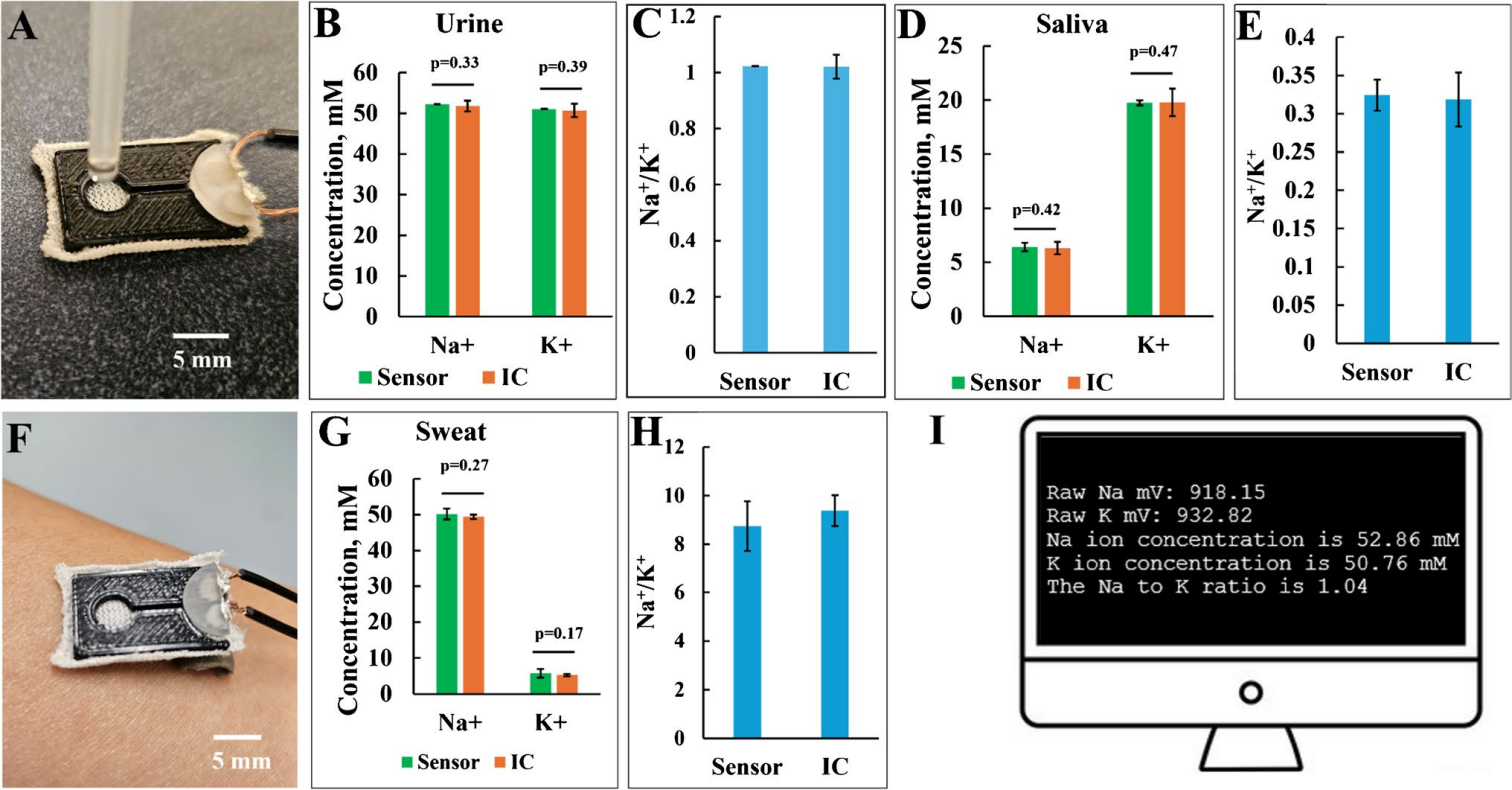
Real applications of the sensor to detect Na^+^ and K^+^ in various biofluids. **A **How a urine or saliva sample could be loaded to the sensor (100 µL sufficient to fill the whole fluidic space). **B** Urinary Na^+^ and K^+^ levels detected by the sensor (green bars) and the validating ion chromatography (IC) method (orange bars). *N* = 3, error bar = standard deviation. **C** The calculated Na^+^/K^+^ ratio in the urine sample. *N* = 3, error bar = standard deviation. **D** Saliva Na^+^ and K^+^ levels detected by the sensor (green bars) and the validating ion chromatography (IC) method (orange bars). *N* = 3, error bar = standard deviation. **E** The calculated Na^+^/K^+^ ratio in the saliva sample. *N* = 3, error bar = standard deviation. **F** Tilting the device to let the circular reservoir part wick sweat from skin. **G** Sweat Na^+^ and K^+^ levels detected by the sensor (green bars) and the validating ion chromatography (IC) method (orange bars). *N* = 3, error bar = standard deviation. **H** The calculated Na^+^/K^+^ ratio in the sweat sample. *N* = 3, error bar = standard deviation. **I** An example of what results were presented by the programmed Arduino on a computer screen

We tested three common biofluids with Na^+^ and K^+^ concentrations highly varied in physiology: urine, saliva, and sweat. Physiological Na⁺ and K⁺ levels vary widely across biofluids—with sweat containing approximately 20–80 mM Na⁺ and 4–8 mM K⁺, saliva containing roughly 5–20 mM Na⁺ and 15–30 mM K⁺, and urine containing about 40–220 mM Na⁺ and 20–90 mM K⁺—all of which fall within the dynamic range of our sensors (5–500 mM) [7, 58–60]. Figure 5A shows how urine and saliva samples could be loaded into the sensor. For urine tests (Fig. 5B), the sensor results (green bars) were not significantly different from ion chromatography validations (orange bars). We also programmed the Arduino to calculate the Na^+^/K^+^ ratio as a readout. As shown in Fig. 5C, the urinary Na⁺/K⁺ ratio was approximately 1.05 from both the sensor and ion chromatography results, and the ratio should be higher than 1 in healthy human [59]. In saliva testing (Fig. 5D), Na⁺ (6.40 ± 0.39 mM, mean ± stdev) and K⁺ (19.74 ± 0.23 mM, mean ± stdev) levels were lower than in urine, consistent with physiology [61]. The sensor readouts and the ion chromatography validation measurements were also highly consistent. Figure 5E shows that the Na⁺/K⁺ ratio was approximately 0.35 in saliva, where potassium does typically exceed sodium (physiological Na⁺/K⁺ ratio 0.3–0.6) [60]. Sweat testing was different in sample loading. As demonstrated in Fig. 5F, the device was placed on a skin surface slightly tilted, with the microfluidic reservoir touching a sweating area to wick and then deliver the sweat through the channel. Figure 5G shows the detected Na⁺ (50.20 ± 1.48 mM, mean ± stdev) and K⁺ (5.74 ± 0.64 mM, mean ± stdev) by the sensor (green bars) and by ion chromatography (orange bars). Figure 5H shows the Na⁺/K⁺ ratio was approximately 10, which was consistent with human physiology (ratio 8–12) because sodium is the mostly excreted cation into sweat with potassium needs to be retained within the body [58, 62]. Figure 5I is an example screen-print (fitted into a screen-frame icon for easier demonstration) of a measurement, with detected potentials/voltages converted to Na^+^ and K^+^ concentrations via built-in calibration curve data, and the Na^+^/K^+^ ratio presented; and the concentration of Na^+^ and K^+^ could be detected and data processed simultaneously with two sensor setups connected to the multiple ports of an Arduino board (typically 6–18 of such ports depending on the board type).

### Expanded discussion: can the microfluidic devices be directly 3D-printed onto fabric substrates?

During FDM 3D-printing, a filament (e.g., ABS) is melted at the printing nozzle, and the molten material writes on the printer stage layer by layer. We then asked whether it was feasible to directly print ABS microfluidics onto a fabric immobilized on the printer stage, with molten ABS infiltrating into the fabric to form hydrophobic areas before it hardened, which could further simplify the sensor fabrication process for mass-producing. As shown in Figure S4 in the SI, after optimizing the printing nozzle and stage temperatures, the printing speed, and the nozzle size to 400 µm orifice, and recalibrating the stage height, the microfluidic devices could be printed onto a piece of cotton fabric. Nonetheless, liquid delivery issues could be observed. For instance, in the specific printing shown in Figure S4C in the SI, one fluidic channel was blocked, two of the six devices had larger reservoirs than the design size, indicating poor formation of the surrounding hydrophobic barriers, and one of the devices had a shrunk reservoir. This data suggests that the method may not be sufficient to reproducibly produce the microfluidic-on-fabric devices yet but can be expected to be improved when more advanced FDM printers are available.

## Conclusion

We have demonstrated a robust and reusable 3D-printed microfluidic-on-fabric sensor with modular electrodes for POC diagnosis. The sensor platform itself represents a new technology for POC sensing applications, and the optimal conditions were determined to form well-defined microfluidic channels and efficient liquid transport, while the modular design enabled customizable sensing. We showcased the application of the sensor platform for Na⁺ and K⁺ detections, which achieved robust analytical performance with high selectivity, reproducibility, and Nernst sensitivity. Moreover, the sensors demonstrated superior reusability after rinsing, with no carryover issues during repeated use. Successful application to urine, saliva, and sweat confirmed the device’s ability to monitor Na⁺ and K⁺ from various biofluids. Together, these results highlight the potential of this sensor platform for POC electrolyte monitoring and possibly broader health diagnostics.

## Supplementary Information

Below is the link to the electronic supplementary material.

Supplementary file1 (DOCX 1.42 MB)

## Data Availability

All data are presented in the manuscript and the supplementary information.
