# Supplementary material for: Reusable 3D-printed microfluidic-on-fabric with modular electrodes for point-of-care Na+ and K+ detection in various biofluids
Source: Anal Bioanal Chem. 2026 May 22;418(14):4651–61. doi: 10.1007/s00216-026-06569-0 (PMC13375825; doi:10.1007/s00216-026-06569-0)
Supplement: Supplementary file 1 — Supplementary file1 (DOCX 1.42 MB) [file 216_2026_6569_MOESM1_ESM.docx]

**Supplementary Information**

**Reusable 3D-Printed microfluidic-on-Fabric with Modular Electrodes for Point-of-Care Na^+^ and K^+^ Detection in Various Biofluids**

Tao Zhang^1,2^, Grayson Ruffner^1^, Sarah Varney^2^, Inchan Baek^3^, Bella Fong^2^, Hui Chen^2,^* and Chengpeng Chen^1,^*

1. Department of Chemistry and Biochemistry,

University of Maryland Baltimore County,

MD, USA, 21250

1. Department of Chemistry and Biochemistry,

James Madison University,

VA, USA, 22801

1. Phillips Academy,

MA, USA, 01810

*Corresponding to: Dr. Chengpeng Chen

1000 Hilltop Circle, Baltimore, MD, USA

Chemistry and Biochemistry

University of Maryland Baltimore County

+1-4104553053

cpchen@umbc.edu

^*Co-corresponding to: Dr. Hui Chen^

901 Carrier Drive

Chemistry and Biochemistry

James Madison University

+1-6319949866

chen22hx@jmu.edu

**
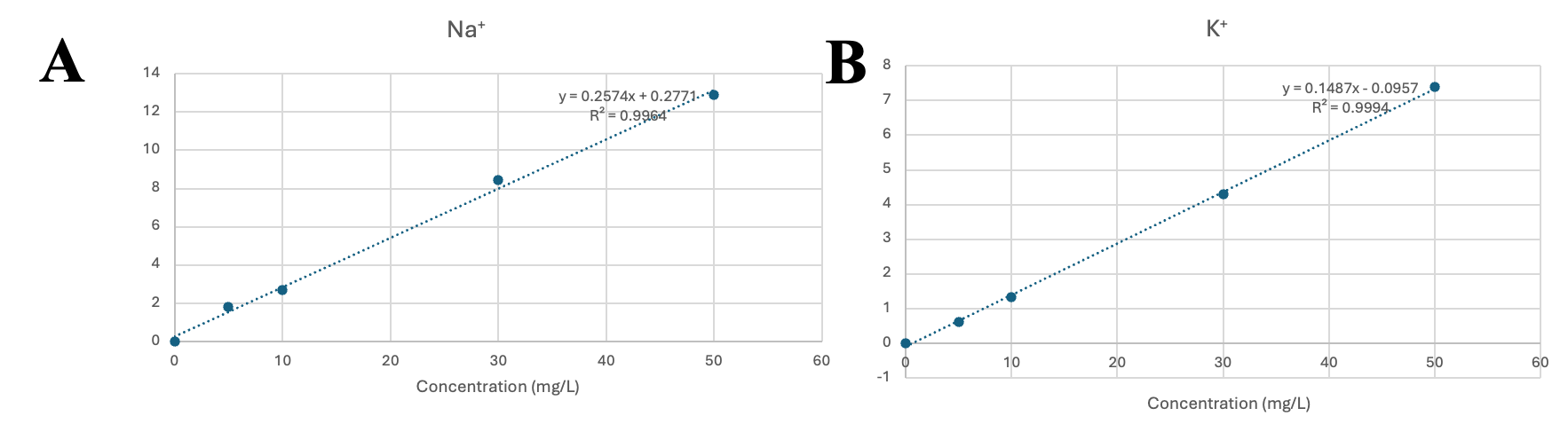
**

**Figure S1.** (A) An example calibration curve for Na⁺ obtained using ion chromatography. (B) An example calibration curve for K⁺ obtained using ion chromatography. Y-axis was normalized peak area.

**
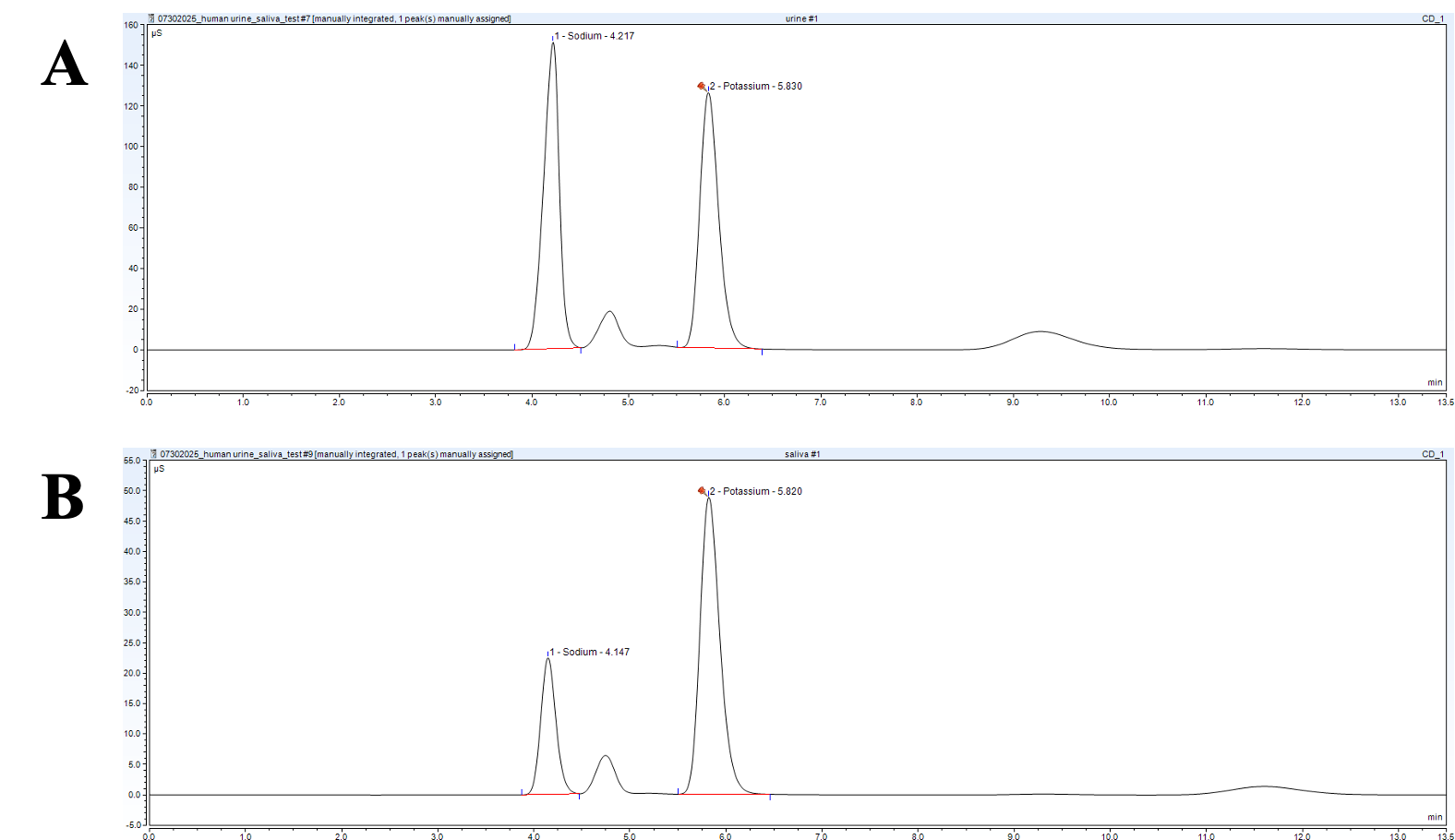
**

**Figure S2.** (A) An example chromatogram of a urine sample obtained using ion chromatography. (B) An example chromatogram of a saliva sample obtained using ion chromatography.

**
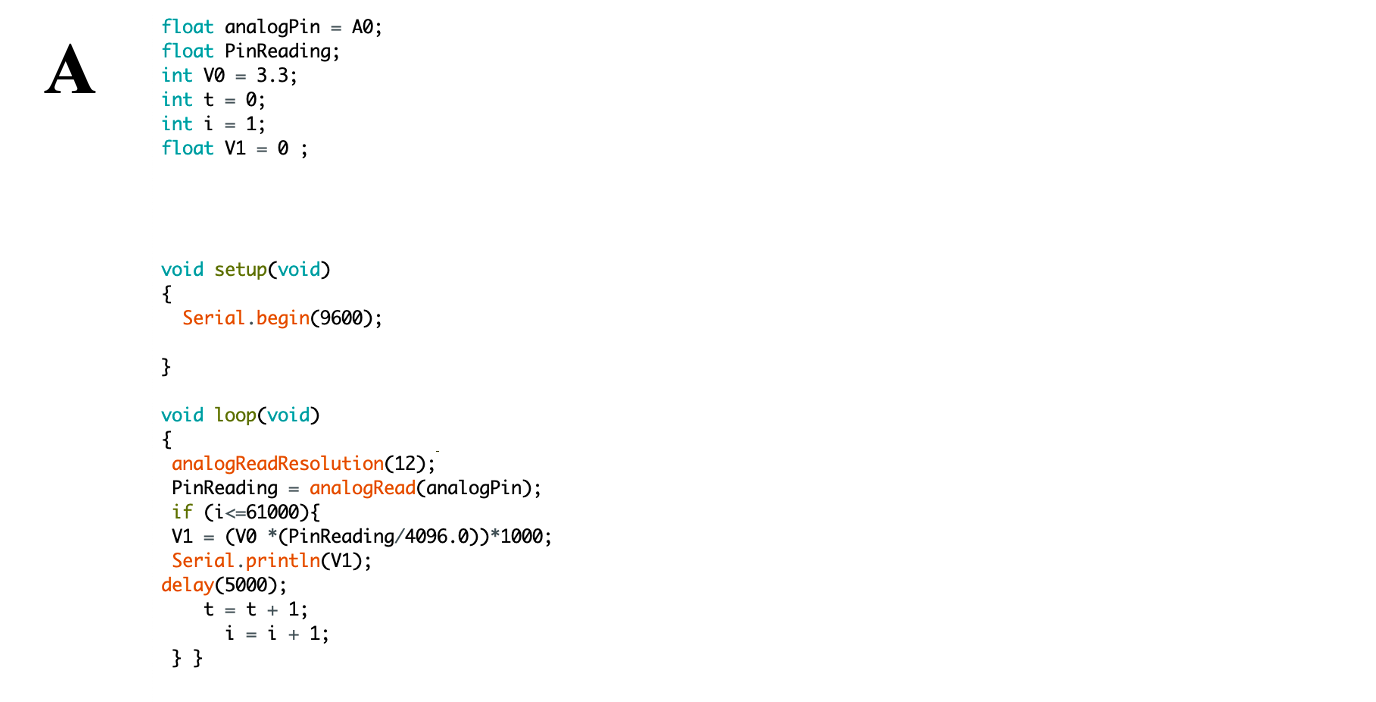
**

**
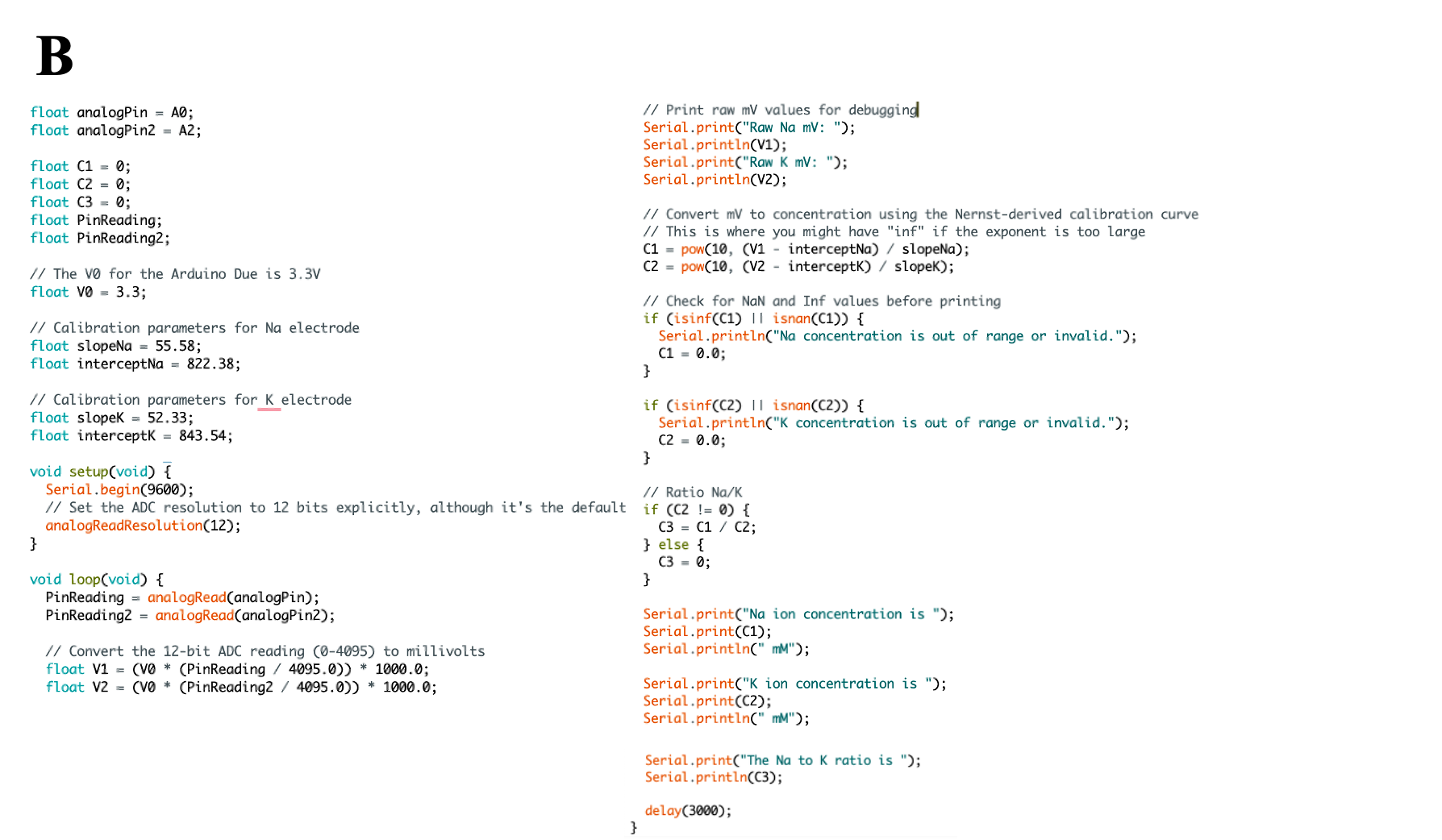
**

**Figure S3.** (A) The Arduino code for voltage reading. (B) The Arduino code for Na^+^ and K^+^ transducing.

**^
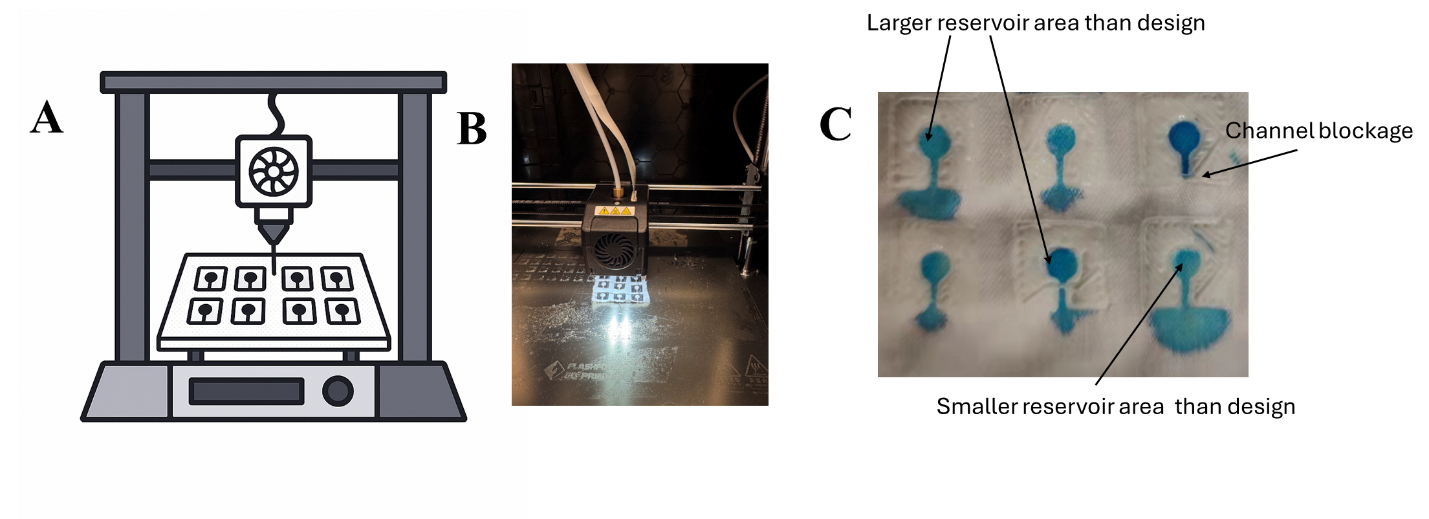
^**

**Figure S4.** (A) Cartoon illustration showing the microfluidic printing process. (B) Photograph of direct printing onto a fabric substrate. (C) Image of the directly 3D-printed microfluidics-on-fabric with a blue dye solution flowing from the reservoir into the channel.
